# Understanding the relationship between costs and the modified Rankin Scale: A systematic review, multidisciplinary consensus and recommendations for future studies

**DOI:** 10.1177/2396987316684705

**Published:** 2017-03-01

**Authors:** Alastair Wilson, Philip MW Bath, Eivind Berge, Dominique A Cadilhac, Matthieu Cuche, Gary A Ford, Rachael Macisaac, Terence J Quinn, Matthew Taylor, Matthew Walters, Claudia Wolff, Kennedy R Lees, for the ESO-VISTA Health Economics Working Group

**Affiliations:** 1 Institute of Cardiovascular and Medical Sciences, University of Glasgow, Queen Elizabeth University Hospital, Glasgow, UK; 2 Stroke Trials Unit, Division of Clinical Neuroscience, University of Nottingham, City Hospital Campus, Nottingham, UK; 3 Department of Internal Medicine, Oslo University Hospital, Oslo, Norway; 4 School of Clinical Sciences, Monash University, Clayton, Victoria, Australia; 5 Medtronic, Tolochenaz, Switzerland; 6 Oxford Academic Health Science Network, Magdalen Centre North, Oxford Science Park, Oxford, UK; 7 Institute of Cardiovascular and Medical Sciences, University of Glasgow, Glasgow Royal Infirmary, Glasgow, UK; 8 York Health Economics Consortium, University of York, York, UK; 9 Institute of Cardiovascular and Medical Sciences, University of Glasgow, Glasgow, UK

**Keywords:** Health economics, modified Rankin Scale, stroke, systematic review

## Abstract

**Background and purpose:**

Cost-of-illness studies often describe a single aggregate cost of a disease state. This approach is less helpful for a condition with a spectrum of outcomes like stroke. The modified Rankin Scale is the most commonly used outcome measure for stroke. We sought to describe the existing evidence on the costs of stroke according to individual modified Rankin Scale categories. This may be useful in future cost effectiveness modelling studies of interventions where cost data have not been collected, but disability outcome is known.

**Methods:**

Systematic review of the published literature, searching electronic databases between 2004 and 2015 using validated search filters. Results were screened to identify studies presenting costs by individual modified Rankin Scale categories.

**Results:**

Of 17,782 unique identified articles, 13 matched all inclusion criteria. In only four of these studies were costs reported by modified Rankin Scale categories. Most studies included direct medical costs only. Societal costs were assessed in two studies. Overall, studies had a high methodological and reporting quality. The heterogeneity in costing methods used in the identified studies prevented meaningful comparison of the reported cost data. Despite this limitation, the costs consistently increased with greater severity (increasing modified Rankin Scale score).

**Conclusions:**

Few cost studies of stroke include information based on stroke recovery measured by individual modified Rankin Scale categories and the existing data are limited. To reliably capture this information, future studies are needed that preferably apply standardised costing methods to promote greater potential for use in cost-effectiveness analyses whereby direct collection of patient-level resource use has not been possible.

## Introduction

Stroke is expensive in terms of its personal, societal and financial impact. The clinical benefit of stroke treatments is usually evaluated according to the functional outcome measures assessed at least three months after stroke, when most of the acute recovery has occurred. The spectrum of stroke outcomes can be assessed using the mRS,^1,2^ which is the most prevalent outcome measure in published trials across recent decades. The 90-day mRS is also the recommended primary outcome measure in acute stroke trials by the European Stroke Organisation (ESO) Outcomes Working Party.^1,2^

The treatment of stroke is complex and costly with effective treatments including stroke unit care, intravenous thrombolysis with recombinant tissue plasminogen activator and most recently, thrombectomy using stent retriever devices.^3,4^ Implementation of new treatments requires the assessment of both cost and outcomes in relation to alternative available interventions or current practice using cost-effectiveness analyses. Reliable cost data relating the mRS by category would be valuable to the wider stroke community when undertaking these forms of health economic evaluations.

The collection of robust data for economic evaluations may be complex and time-consuming, increasing the expense of trials. Therefore, to include cost-effectiveness evaluations as part of stroke trials can be challenging, and add to responder burden through the need for additional questionnaires. Quantifying the cost of a chronic, disabling condition such as stroke is complicated since, to provide a full picture of the cost impact to society, it is important to capture the direct costs of hospital care, as well as the direct and indirect costs over the longer term, including lost productivity. Having reliable estimates of costs by functional outcome that could be applied in cost-effectiveness studies would facilitate the ability of investigators to perform these important evaluations more often.

We have undertaken a systematic review of the current literature investigating the relationship between costs of stroke and functional outcome as measured by the mRS as a basis for informing the field and understanding the evidence base that may be available for cost-effectiveness evaluations where mRS data have been captured. Through the assessment of the literature, the ESO aims to eventually develop practical guidance for the integration of health economic data collection in future studies. By identifying and reporting current information on the costs for each mRS category, these could then be applied in decision-analytic simulations or estimations of the potential cost-effectiveness of new interventions in stroke, where primary collection of cast data has not been possible.

## Methods

We performed a systematic review of the published literature on studies where the costs of stroke by mRS category were reported. To guide the systematic review, we applied the principles of the PRISMA statement (Appendix 2). We reviewed publications from 1 January 2004 to 13 February 2015 in the following electronic databases: MEDLINE (Ovid); EMBASE (Ovid); PsychINFO (EBSCO); CINAHL (EBSCO) and National Health Service Economic Evaluation Database (NHS EED).

A sensitive search strategy was designed to incorporate two concepts, (1) Stroke and (2) Health economics, which were linked using the Boolean operator ‘AND’. We developed the Concept 1 strategy using guidance from the Cochrane Stroke Group and the strategy for Concept 2 using NHS Centre for Review and Dissemination (CRD) economic study search guidelines. Terms were tailored to each database taking into account unique topic headings and syntax. We also applied a Concept 3 utilising pre-coordination of information retrieval. This permits direct access to topic results using Emtree or MeSH subheadings e.g. Stroke/ec [Economics] for MeSH in Medline and cerebrovascular accident/dm [Disease Management] for Emtree in EMBASE. The results of our Concept 1 and Concept 2 searches were linked to Concept 3 by search operator ‘OR’. Appendix 1 shows the detail of the search strategies for all databases, and any limitations that applied to the results by author AW.

Duplicate results were filtered out using EndNote reference manager (version X7.2.1, Thomson Reuters, USA) and citations were screened by title for relevance. We also filtered out citations that referred only to conference proceedings or abstracts before screening citations by title for their relevance.

The following inclusion and exclusion criteria were applied to title/abstract review of relevant search results:

### Inclusion

1. Adult (18+).
2. Includes costs data (indirect and/or direct costs reported i.e. hospital stay, carer, medications, loss of workplace earnings, etc. were all eligible).
3. Acute stroke.
4. mRS reported as the health outcome.

### Exclusion

1. Subarachnoid haemorrhage or traumatic brain injury.
2. Protocols or methodologies for randomised controlled trials (RCT).
3. Cost-effectiveness studies comparing one or more intervention.

We assessed the included studies for reporting and methodological quality. Currently, there is no consensus on the best instrument for assessing the methodological and reporting quality of cost-of-illness studies. In this review, we followed the recommendation of Cochrane handbook and utilised the checklist developed by Drummond and Jefferson,^5^ as relevant to cost-of-illness studies This focuses on three domains: study design; data collection and analysis; and interpretation of results. This checklist can be applied to range of health economic designs encompassing both full cost-effectiveness studies and cost-of-illness studies.

The costs from the included studies were abstracted and then converted to relative 2015 costs in Euros accounting for inflation to allow for direct comparison of the results. Purchasing power parity (PPP) was used to calculate the relative value to each currency. Germany was chosen as having the most representative healthcare system and economy, and provided the ‘baseline’ Euro currency from which to calculate the PPP. The calculations were performed using a web-based calculator developed by Campbell and the Cochrane Economics Methods Group in conjunction with the Evidence for Policy and Practice Information and Coordinating Centre.^6,7^

Our aims were to present an estimate of cost of illness relative to stroke severity as measured by the mRS. However, given the recognised heterogeneity in methods used in health economic studies such as cost-of-illness studies,^8^ where we were unable to make any meaningful comparison among studies, we have presented a narrative review of the findings.

## Results

**Figure 1.**
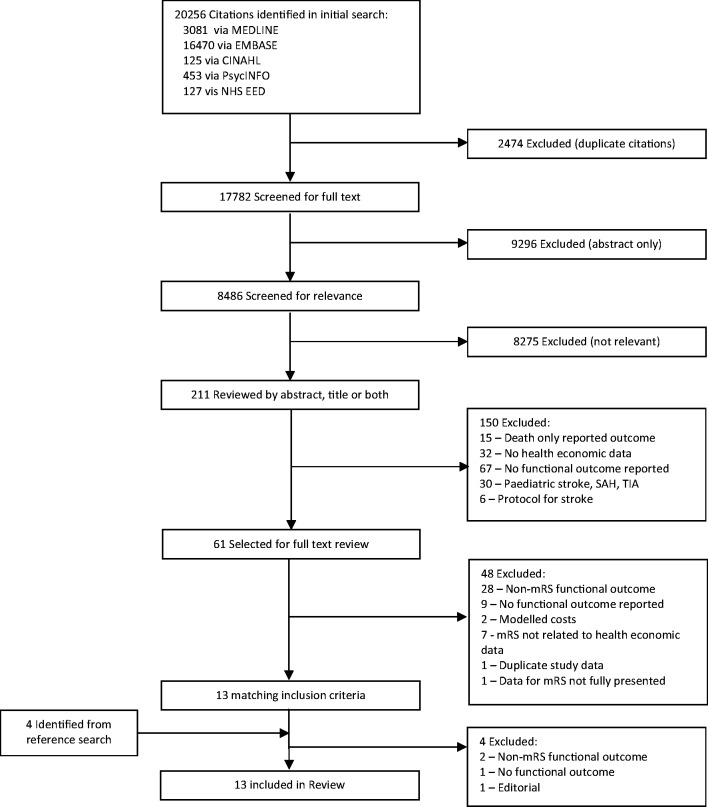
Result of systematic search strategy.

The literature search yielded 8486 unique full text articles that were screened for inclusion in the study (Figure 1). From these, we identified and selected 61 relevant studies for full text review. Of these, only 13 met the inclusion criteria and have been included for reporting in this review. The characteristics of the selected studies are shown in Table 1. We included one study^9^ that had reported costs by individual mRS categories as part of a nested cohort study, whereby these cost-of-illness estimates were then later applied in a cost-effectiveness analysis of thrombolysis treatment.

**Table 1.** Characteristics of included studies.

Studies	Number of Patients	Country	Time frame	Currency	Follow up	Additional info.	Average total cost per mRS (SD)						
							0	1	2	3	4	5	6
Asil et al.^10^	328	Turkey	January 2007– December 2007	USD	Discharge		1000 (729)			1838 (1895)			2777 (5792)
Baeten et al.^11^	338	Netherlands	January 1999– January 2000	Euro	6 months	Stroke service	8400		11,080		29,644	27,371	
					6 months	Usual Care	9856		14,868		37,682	46,089	
					12 months	Usual Care	1761		4196		17,824	22,515	
Christensen et al.^12^	820	Worldwide	May 2005– February 2007	USD	3 months		9466 (4614)	15,547 (7873)	18,742 (9978)	27,387 (11,621)	27,281 (8,919)	27,330 (11,495)	8136 (3719)
						(95% CI)	7130– 11,902	13,336– 17,757	15,987– 21,496	24,372– 30,402	25,198– 29,364	22,182– 32,479	7421–9032
Christensen et al.^13^	167	Argentina	January 2004– August 2006	USD	Discharge	ICH	1475				6370		
					Discharge	IS	1622				3188		
Christensen et al.^14^	316	Brazil	January 2006– May 2007	USD	Discharge	ICH	6307					30,693	
					Discharge	IS	1800				6771		
Dawson et al.^15^	1717	Worldwide	March 1998– May 1999	GBP	3 months		2493 to 3412	3369 to 4479	5784 to 7008	7300 to 8515	10,095 to 11,141	11,772 to 13,560	
Dodel et al.^16^	340	Germany	January 2000– June 2000	Euro	Discharge		3160 (1300)			4030 (2780)			
Epifanov et al.^17^	253	Germany	January 1998– June 1998	Euro	Discharge		3,210 (2170)			3350 (3050)			
Fattore et al.^18^	411	Italy	August 2005– March 2007	Euro	0–3 months		1225			3370	9185		
					3–6 months		343			1688	2335		
					6–12 months		513			939	2182		
					12 month total		1964			5239	13,381		
Hayes et al.^19^	172	USA	May 2001– September 2002	USD	3 months		7044	6159	7885	11,723	22,156	18,670	
					3–12 months		4321	5727	3994	6811	5154	9958	
					12 month total		14,901	12,637	12,751	23,218	30,971	28,628	
Luengo-Fernandez et al.^20^	153	United Kingdom	April 2002– March 2007	GBP	3 months		3945 (7558)			17,406 (18,417)		25,279 (16,396)	
					Annual costs		2135 (3675)			4165 (7668)		6234 (14,898)	
Spieler et al.^21^	435	France	Unavailable	Euro	18 months		10,255			17,457	31,728		
						(95% CI)	9679–10,831			14,46– 020,453	28,811–34,645		
Tanny et al.^9^	378	Australia	January 2003– December 2011	USD	3 months		29,406	32,214	36,205	37,878	39,522	41,780	16,727

Note: Tan Tanny et al, calculated hospital costs from 378 patients based on actual expenditure sourced from the Clinical costing unit of Royal Melbourne Hospital and inputted these costs in the cost effectiveness model of thrombolysis treatment.

### Description of included studies

Among the articles that we identified, the authors had investigated populations from diverse locations. Six studies were European (46%) and two were worldwide multicentre trials.^13,15^ Costs were quoted in three currencies: US dollars, Euros and Pounds Sterling. A broad range of methods had been used to determine costs in these currencies, but most had applied PPP to establish a common value to each currency worldwide. Patient data collection for the included studies was conducted from March 1998^15^ through December 2011.^9^ Eleven studies reported costs up to 90 days (84%); and in five studies, the longer term costs of stroke of between 6 to 18 months were reported.^11,18–21^

### Quality assessment

The application of the Drummond et al.^5^ checklist to the studies shows the overall quality of the study was high (Table 4). However, presentation of results in both aggregate and disaggregate forms was handled poorly by the authors of these studies. Only 30% presented results as full ordinal mRS in relation to costs.

### Cost of stroke by mRS category

Table 1 shows the total cost of stroke by mRS grade alongside any measures of uncertainty. The data collected from the studies are heterogeneous, with diverse resources recorded and included in the overall total cost per mRS grade (Table 2).

**Table 2.** Perspective and resources collected in identified studies.

Studies	Perspective	Index hospitalisation costs		Post-acute resources					Thrombolysis included in costs
		Length of stay^a^	Direct medical costs^b^	Professional appointments^c^	Rehabilitation appointments^d^	Home health care^e^	Productivity loss^f^	Length of stay^g^	
Asil, et al.^10^	Hospital	✓	✓						8 patients in cohort
Baeten^11^	Healthcare	✓	✓	✓	✓			✓	Not included
Christensen and Morris^12^	Healthcare	✓	✓						Not included
Christensen et al.^13^	Healthcare	✓	✓						Not included
Christensen et al.^14^	Healthcare	✓	✓	✓	✓	✓			Not included
Dawson et al.^15^	Hospital	✓							Not included
Dodel et al.^16^	Hospital	✓	✓						Included in cohort
Epifanov et al.^17^	Hospital	✓	✓						Not stated
Fattore et al.^18^	Societal	✓	✓	✓	✓	✓	✓		Not stated
Hayes et al.^19^	Hospital	✓	✓	✓	✓			✓	Not stated
Luengo-Fernandez et al.^20^	Healthcare	✓	✓	✓					Not stated
Spieler et al.^21^	Societal	✓	✓	✓	✓	✓	✓		Not stated
Tanny et al.^9^	Hospital	✓	✓					✓	All patients in cohort

Note:

^a^Stroke unit, ER, ICU, General ward, intermediate care facility, rehabilitation facility, nursing/convalescence home.

^b^Imaging, diagnostic tests, laboratory tests, surgical interventions and drug costs.

^c^General practitioner visits, emergency care, outpatient visits.

^d^Physiotherapy, speech therapy, ergo therapy.

^e^Paid home healthcare, informal care, home adaptation, ortheses.

^f^Loss of working days.

^g^Rehabilitation facility, nursing/convalescence home.

Table 3 shows all mRS scores aggregated with associated costs in a common currency (Euro) adjusted for inflation and PPP and presented according to the time of assessment. The range of costs reported for mRS 1 is €1614 to €26,079 and for mRS 4: €4,754 to €35,050. The evaluated studies represent a range of follow-up time points at which the costs were recorded. The majority present costs and mRS scores at discharge or at 90 days; but some studies only recorded costs until 10 days or after the initial stroke event or until hospital discharge. In contrast, the studies of Fattore et al.^18^ and Spieler et al.^21^ focussed on longer term time points, reporting mean costs for mRS 3 of €5722 and €21,324 over 12 and 18 months, respectively.

**Table 3.** Costs of Stroke by the mRS scores.

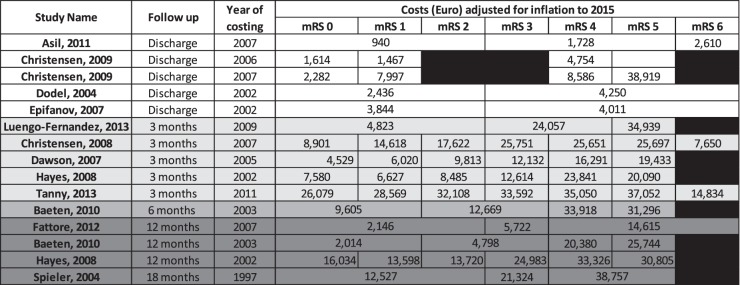	

Note: Costs displayed in Euro adjusted to 2015 using purchasing power parity with Germany as the target currency. All calculations done using CCEMG – EPPI-Centre Cost Converter.^10^, http://eppi.ioe.ac.uk/costconversion/Default.aspx) Table 1 presents costs in original currency at time of study.

**Table 4.** Quality of Included studies assessed by Dummond et al Checklist.

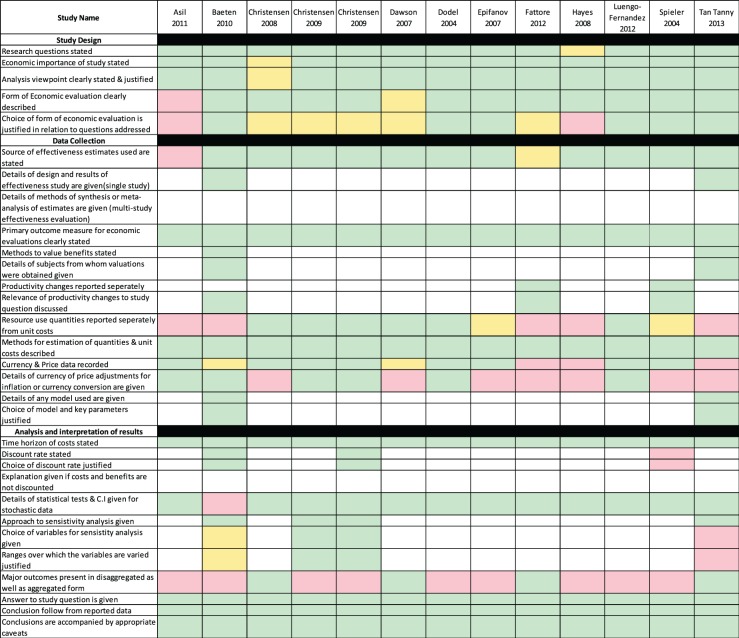	

Note: Colours indicate the level to which the study fulfils criteria; Green – Complete, Yellow – Not clear and Red – Does not fulfil criteria and Blank cells – Category not appropriate to study.

## Discussion

The primary aim of this review was to collate the available data describing the relationship between costs and outcomes based on the mRS scale categories. Establishing a reliable estimation of costs by mRS categories is highly relevant since it may provide an indirect method for undertaking cost-effectiveness analyses of novel interventions to be compared against usual care. This review, however, found that it was not possible to effectively undertake any meaningful analyses due to the heterogeneous nature of the identified studies and lack of long-term follow-up data.

We identified 13 studies incorporating cost of stroke relating to an mRS score; only three studies provided an estimate of people who later died from stroke (mRS 6). However, there was significant methodological heterogeneity which precluded the ability to make any meaningful comparison between the stated costs either at a single mRS category or across the scale. Tables 1 and 2 highlight this heterogeneity, showing the diversity in time horizon (30–540 days), included resources and study perspectives.

The time horizon for the collection of costs in these studies will have a large influence on the overall costs associated with stroke. Among the 13 studies identified, five recorded costs up until discharge, five at 90 days and five included costs for longer time frames (6 to 18 months) post stroke. Baeten et al.^11^ and Hayes et al.^19^ included costs at multiple time points. Costs in stroke are highly dependent on the time of collection with the intervention, rehabilitation and associated hospital costs concentrated in the acute phase (up to 90 days), while longer term costs including home health care, social services assistance, as well as productivity loss are more significant across a broader time period. This is highlighted in the two studies that considered longer term costs. Fattore et al.^18^ and Spieler et al.^21^ provided evidence that direct medical costs were initially high, but quickly plateaued and remained steady after the first 90 days.^21^ However, indirect costs such as productivity losses and paid care increased over time^18^ highlighting the importance of including of capturing costs across a broader time horizon when considering the health economic impact of stroke.

Even when considering the studies that focussed on collecting data from comparable time horizons, there remained a high level of variability between costs reported at each category. This can be accounted for by the heterogeneity in reported resources (Table 2). Of the four studies looking at costs at 90 days using the full ordinal mRS Dawson et al.^15^ and Christensen and Morris^12^ focused on length of stay as their primary cost metric.^22,23^ Additionally, Christensen and Morris^11^also included coverage of rehabilitation and home healthcare costs. Hayes et al.^19^ and Tanny et al.^9^ calculated costs related directly from a patient cohort and extrapolated out of hospital information from relevant local cost-of-illness studies applied to their cohort based on discharge destinations.

There was also a high level of heterogeneity in the reporting of outcomes with only 4 of 13 studies using the mRS as a complete ordinal scale. In other studies, the information on costs by mRS was dichotomised or trichotomised. This latter approach discards valuable information and undermined the ability to undertake meaningful comparisons between the included studies.

To be useful in cost-effectiveness evaluations, the mRS as a measure of functional ability beyond the acute phase of the disease needs to be costed from the perspective of society whereby the direct and indirect costs to the health sector, patients and other sectors, e.g. workforce are captured and summarised. Consistently, the identified studies provided evidence that increasing severity of mRS was associated with increasing direct medical costs. All studies but one^15^ included direct medical costs such as treatment, diagnostic costs and imaging in the estimation of costs at each mRS category. Hospital stay^15^ alone was used as the cost metric in the final study and highlighted the correlation between increased length of stay, mRS severity and increased costs. Capturing finer grained direct medical costs in hospitals is important since a patient who has achieved an mRS of 0 through costly treatment such as thrombectomy^24^ will incur little or no out-of-hospital costs but high direct medical costs. This review has shown that the estimate of costs includes some, if not all of the direct medical costs for the patients care associated with mRS category. However, to allow for comparison and generic estimates to be generated, future studies require more consistency in their methods.

### Strengths and limitations

We employed a comprehensive search strategy utilising validated search strings designed to capture the broadest range possible of available literature investigating both stroke and cost-of-illness studies before combining these themes. The strategy was employed on the four major scientific databases, as well as the NHS EED. This review was carried out using a defined methodological approach to data extraction and critical evaluation of included studies.

Our methods still have limitations. The systematic search and data extraction was carried out by a single author (AW) under the supervision of TQ. The data collected in the review yielded a highly heterogeneous sample based on what was available in the published literature: individual study authors were not approached. Additionally, the scope of this review was focussed on the use of the mRS and did not look at the systematic comparison of trials investigating the Health economics of Stroke using alternative outcome measures.

### ESO Health economics working group meeting 2015

The results of the analysis were presented at the 2015 Health economics workshop at the European Stroke Organisation (ESO) meeting in Glasgow attended by participants from industry and academia. The attendees agreed in principle that standardisation of health economic data collected through clinical trials is required and suggested an international collaboration to develop guidelines for future trials. Attendees at the workshop also noted limited comparability across studies identified within this review, lending further credence to the suggestion that standardisation of resources collected by trialists is required to reduce heterogeneity. The importance of including out-of-hospital direct and indirect costs alongside direct medical costs that are incurred in hospital in future studies was emphasised by workshop members due to the long-term disabling nature of stroke. Attendees also noted that the WHO Research Agenda for Health Economic Evaluation (RAHEE) project in Stroke is working towards similar aims and could be approached for collaboration.^25^

Working groups to develop these guidelines have been assembled and the development of a prospective study investigating resource use in stroke trials is being undertaken.

### Summary of suggested guidelines for future trials

The result of the systematic review has yielded a four-point list of suggested guidelines for stroke researchers to optimise the collection of health economic information in future trials which are summarised below.

1. Resource use and mRS to be collected at 90 days post stroke*.
2. mRS to be presented as a complete ordinal scale to preserve information relating to costs including those for patients who later died (mRS 6)*.
3. Collection of resources used to be standardised. To this end, it is proposed that a group such as the ESO Economics Working Group develop a template and recommended costing methods as a resource to support this activity.
4. Presentation of cost analyses to include measures of variability allowing for meta-analysis of aggregate data.

*As recommended by the European Stroke Organisation (ESO) Outcomes Working Party.

## Conclusion

Our findings have provided a valuable insight into the heterogeneity seen in health economic reporting in the field of stroke, in particular for the most commonly collected stroke outcome measure used in trials, the mRS. This heterogeneity undermined the meaningful comparison of the included studies and until further data are available for systematic analysis, we recommend readers refer to the original source data when assessing critical quality and relevance to ongoing research. It has also outlined a need for more real world and trial data investigating health economic outcomes in stroke looking at both short and long-term costs related to the mRS as an ordinal scale. This work has provided a foundation from which to address the need for the development of guidelines for health economic data and promotion of its importance amongst current and future trialists in the area of stroke.

## Supplementary Material

Supplementary material

Supplementary material

## ESO-VISTA Working Group

Dr. Myzoon Ali – University of Glasgow, Prof. Philip MW Bath – University of Nottingham, Dr. Eivind Berge – University of Oslo, Anne-Laure Bocquet – Stryker, Prof. Natan Bornstein – Tel Aviv Sourasky Medical center, Prof. Marian Brady – Glasgow Caledonian University, Chloe Brown – Neuravi, Assoc. Prof. Dominiqe Cadilhac – Monash University, Dr. Bruce Campbell – Royal Melbourne Hospital, Assoc. Prof. Hanne Christensen – University of Copenhagen, Alain Cornil – Penumbra, Matthieu Cuche – Covidien, Daniel d Atillio – Penumbra, Avinoam Dayan – Brainsgate, Edith Doppler – Ever NeuroPharma, Prof. Gary Ford – University of Oxford, Dr. Rachael Fulton – University of Glasgow, Prof. Markku Kaste – University of Helsinki, Dr. Matthew Leathley – University of Central Lancashire, Prof Kennedy R Lees – University of Glasgow UK, Noam Levy – Brainsgate, Assoc. Prof. Atte Meretoja – University of Melbourne, Dr. Patrik Michel – University of Lausanne, Natalie Mühlemann – Nestle, Marine Provoyeur – Penumbra, Stacey Pugh – Covidien, Dr. Terry Quinn – University of Glasgow, Prof. Jeffrey Saver – UCLA, Dr Jan-Friedrich Scheitz – Charité – Universitätsmedizin Berlin, Dr. Peter Schellinger – John Wesling Medical Center Minden, Lauren Sheppard – Deakin University, Yoram Solberg – Brainsgate, Assoc. Prof. Nikola Sprigg – University of Nottingham, Dr Matthew Taylor – York Health Economics Consortium, Dr. Götz Thomalla – University of Hamburg-Eppendorf, Prof. Matthew Walters – University of Glasgow, Prof. Steven Warach – University of Texas Southwestern Medical Center, Prof. Joanna Wardlaw – University of Edinburgh, Prof. Christian Weimer – University of Essen, Dr. Alastair Wilson – University of Glasgow, Claudia Wolff – Medtronic
