# Supplementary material for: Understanding the relationship between costs and the modified Rankin Scale: A systematic review, multidisciplinary consensus and recommendations for future studies
Source: Eur Stroke J. 2017 Mar 1;2(1):3–12. doi: 10.1177/2396987316684705 (PMC5992734; doi:10.1177/2396987316684705)
Supplement: Supplementary material [file ESO684705_supplementary_material1.pdf]

Supplementary Materials

Appendix 1: ESO workshop search strategy

All searches conducted 2004-Current.

"Concept 1" = Stroke

Search string derived from validated search terms by Cochrane Stroke Group

OVID/MEDLINE: Ovid MEDLINE(R) In-Process & Other Non-Indexed Citations and Ovid MEDLINE(R) 1946 to Present

1	cerebrovascular disorders/
2	exp basal ganglia cerebrovascular disease/
3	exp brain ischemia/
4	exp carotid artery diseases/
5	exp intracranial arterial diseases/
6	exp intracranial embolism and thrombosis/
7	exp intracranial he?morrhages/
8	exp stroke/
9	exp brain infarction/
10	exp vertebral artery dissection/
11	1 or 2 or 3 or 4….or 10
12	stroke .ti,ab,kf
13	cerebrovasc$ .ti,ab,kf
14	cerebral vascular accident$ .ti,ab,kf
15	brain vasc$ .ti,ab,kf
16	cerebral vasc$ .ti,ab,kf
17	cva$ or apoplex$ .ti,ab,kf
18	(cerebral arteriovenous malformation$ or AVM or avms) .ti,ab,kf
19	middle cerebral artery .ti,ab,kf
20	aneurysm$ .ti,ab,kf
21	carotid .ti,ab,kf
22	"cerebro vasc$" .ti,ab,kf
23	11 or 12 or 13….or 22
24	brain$ .ti,ab,kf
25	cerebr$ .ti,ab,kf
26	cerebell$ .ti,ab,kf
27	vertebrobasilar .ti,ab,kf
28	hemispher$ .ti,ab,kf
29	intracran$ .ti,ab,kf
30	intracerebral .ti,ab,kf
31	infratentorial .ti,ab,kf
32	supratentorial .ti,ab,kf
33	MCA .ti,ab,kf

34 anterior circulation .ti,ab,kf
35 posterior circulation .ti,ab,kf
36 basal ganglia .ti,ab,kf
37 24 or 25 or 26….or 37
38 isch?emi$ .ti,ab,kf
39 infarct$ .ti,ab,kf
40 thrombo$ .ti,ab,kf
41 emboli$ .ti,ab,kf
42 39 or 40 or 41 or 42
43 (39) adj5 (43)
44 brain$ .ti,ab,kf
45 cerebr$ .ti,ab,kf
46 intracran$ .ti,ab,kf
47 intraventricular .ti,ab,kf
48 infratentorial .ti,ab,kf
49 supratentorial .ti,ab,kf
50 basal ganglia .ti,ab,kf
51 subarachnoid .ti,ab,kf
52 parenchymal .ti,ab,kf
53 45 or 46 or 47…53
54 haemorrhage$ .ti,ab,kf
55 hemorrhage$ .ti,ab,kf
56 haematoma$ .ti,ab,kf
57 hematoma$ .ti,ab,kf
58 bleed$ .ti,ab,kf
59 55 or 56 or 57 or 58 or 59
60 (54) adj5 (60)
62 poststroke . ti,ab,kf
63 Post$stroke .ti,ab,kf
64 isch?emi$ attack$ .ti,ab,kf
65 tia$1 .ti,ab,kf
66 neurologic$ deficit$ .ti,ab,kf
67 SAH .ti,ab,kf
68 AVM .ti,ab,kf
69 62 or 63 or 64… or 70
70 11 or 23 or 44 or 61 or 71

OVID/EMBASE: Embase 1996 to 2015 Week 06

1 cerebrovascular disease/
2 basal ganglion hemorrhage/
3 exp brain hematoma/
4 exp brain hemorrhage/
5 exp brain infarction/
6 exp brain ischemia/
7 exp carotid artery disease/
8 cerebral artery disease/
9 cerebrovascular accident/
10 exp intracranial aneurysm/
11 exp occlusive cerebrovascular disease/
12 stroke/
13 1 or 2 or 3… or 12
14 stroke patient/
15 Stroke unit/
16 14 or 15
17 stroke. ti,ab,kw.
18 cerebrovasc$. ti,ab,kw.
19 brain vasc$. ti,ab,kw.
20 cerebral vasc$. ti,ab,kw.
21 cva$. ti,ab,kw.
22 apoplex$. ti,ab,kw.
23 17 or…22
24 brain$. ti,ab,kw.
25 cerebr$. ti,ab,kw.
26 cerebell$. ti,ab,kw.
27 vertebrobasilar. ti,ab,kw.
28 hemispher$. ti,ab,kw.
29 intracran$. ti,ab,kw.
30 intracerebral. ti,ab,kw.
31 infratentorial. ti,ab,kw.
32 supratentorial . ti,ab,kw.
33 MCA. ti,ab,kw.
34 anterior circulation. ti,ab,kw.
35 posterior circulation. ti,ab,kw.
36 basal ganglia. ti,ab,kw.
37 24 or…36
38 isch?emi$ .ti,ab,kw.
39 infarct$ .ti,ab,kw.
40 thrombo$ .ti,ab,kw.
41 emboli$ .ti,ab,kw.
42 38 or 39 or 40 or 41
43 (37) adj5 (42)
44 brain$ .ti,ab,kw

45 cerebr$ .ti,ab,kw
46 intracran$ .ti,ab,kw
47 intraventricular .ti,ab,kw
48 infratentorial .ti,ab,kw
49 supratentorial .ti,ab,kw
50 basal ganglia .ti,ab,kw
51 subarachnoid .ti,ab,kw
52 parenchymal .ti,ab,kw
53 44 or…52
54 haemorrhage$ .ti,ab,kw
55 hemorrhage$ .ti,ab,kw
56 haematoma$ .ti,ab,kw
57 hematoma$ .ti,ab,kw
58 bleed$ .ti,ab,kw
59 54 or 55 or 56 or 57 or 58
60 (53) adj5 (59)
61 poststroke . ti,ab,kw
62 Post$stroke .ti,ab,kw
63 isch?emi$ attack$ .ti,ab,kw
64 tia$1 .ti,ab,kw
65 neurologic$ deficit$ .ti,ab,kw
66 SAH .ti,ab,kw
67 AVM .ti,ab,kw
68 61 or….68
70 13 or 16 or 23 or 43 or 60 or 78

EBSCO/CINAHL

1 Stroke*. [TIAB]
2 Cerebrovasc* . [TIAB]
3 brain vasc*. [TIAB]
4 cerebral vasc*. [TIAB]
5 Cva*. [TIAB]
6 Apoplexy*. [TIAB]
7 1 or…6
8 Brain* . [TIAB]
9 Cerebr*. [TIAB]
10 Cerebell*. [TIAB]
11 vertebrobasilar. [TIAB]
12 Hemispher*. [TIAB]
13 Intracran* . [TIAB]
14 intracerebral. [TIAB]
15 infratentorial. [TIAB]
16 supratentorial . [TIAB]
17 MCA. [TIAB]
18 anterior circulation. [TIAB]

19	posterior circulation. [TIAB]
20	basal ganglia. [TIAB]
21	8 or…20
22	Isch#emi* [TIAB]
23	Infarct* [TIAB]
24	Thrombo* [TIAB]
25	Emboli* [TIAB]
26	22 or 23 or 24 or 25
27	(TI Brain* OR AB Brain* OR TI Cerebr* OR AB Cerebr* OR TI Cerebell* OR AB Cerebell* OR TI vertebrobasilar OR AB vertebrobasilar OR TI Hemispher* OR AB Hemispher* OR TI Intracran* OR AB Intracran* OR TI intracerebral OR AB intracerebral OR TI infratentorial OR AB infratentorial OR TI supratentorial OR AB supratentorial OR TI MCA OR AB MCA OR TI anterior circulation OR AB anterior circulation OR TI posterior circulation OR AB posterior circulation TI basal ganglia OR AB basal ganglia) N6 (TI Isch#emi* OR AB Isch#emi* OR TI Infarct* OR AB Infarct* OR TI Thrombo* OR AB Thrombo* OR TI Emboli* OR AB Emboli*)
28	Brain* . [TIAB]
29	Cerebr* . [TIAB]
30	Intracran* [TIAB]
31	intraventricular [TIAB]
32	infratentorial [TIAB]
33	supratentorial [TIAB]
34	basal ganglia [TIAB]
35	subarachnoid [TIAB]
36	parenchymal [TIAB]
37	28 or… 36
38	H#morrhage* [TIAB]
39	H#matoma* [TIAB]
40	Bleed* [TIAB]
41	38 or 39 or 40 or 41 or 42
42	(TI Brain* OR AB Brain* OR TI Cerebr* OR AB Cerebr* OR TI infratentorial OR AB infratentorial OR TI supratentorial OR AB supratentorial OR TI basal ganglia OR AB basal ganglia OR TI Intracran* OR AB Intracran* OR TI intraventricular OR AB intraventricular OR TI subarachnoid OR AB subarachnoid OR TI parenchymal OR AB parenchymal) N6 (TI H#morrhage* OR AB H#morrhage* OR TI

H#matoma* OR AB H#matoma* OR TI Bleed* OR AB Bleed*)

43
44 Poststroke* [TIAB]
45 post*stroke [TIAB]
46 [TIAB]
47 Isch#emi* attack* [TIAB]
48 Tia*1 [TIAB]
49 Neurologic* deficit* [TIAB]
50 SAH [TIAB]
51 AVM [TIAB]
52 45 or…53
53 7 or 21 or 27 or 44 or 54
54 MW Stroke
55 MH cerebral h#morrhage
56 MM Stroke, Lacunar
57 MM Stroke Volume
58 MM NIH Stroke Scale
59 54 or 55..or 58
60 53 or 59

EBSCO/PsycINFO

1 Stroke*. [TIAB]
2 Cerebrovasc* . [TIAB]
3 brain vasc*. [TIAB]
4 cerebral vasc*. [TIAB]
5 Cva*. [TIAB]
6 Apoplexy*. [TIAB]
7 1 or…6
8 Brain* . [TIAB]
9 Cerebr*. [TIAB]
10 Cerebell*. [TIAB]
11 vertebrobasilar. [TIAB]
12 Hemispher*. [TIAB]
13 Intracran* . [TIAB]
14 intracerebral. [TIAB]
15 infratentorial. [TIAB]
16 supratentorial . [TIAB]
17 MCA. [TIAB]
18 anterior circulation. [TIAB]
19 posterior circulation. [TIAB]

20 basal ganglia. [TIAB]
21 8 or…20
22 Isch#emi* [TIAB]
23 Infarct* [TIAB]
24 Thrombo* [TIAB]
25 Emboli* [TIAB]
26 22 or 23 or 24 or 25
27 (21) N6 (26)
28 Brain* . [TIAB]
29 Cerebr* . [TIAB]
30 Intracran*   [TIAB]
31 intraventricular   [TIAB]
32 infratentorial   [TIAB]
33 supratentorial   [TIAB]
34 basal ganglia   [TIAB]
35 subarachnoid   [TIAB]
36 parenchymal   [TIAB]
37 28 or… 36
38 H#morrhage*   [TIAB]
39 H#matoma* [TIAB]
40 Bleed* [TIAB]
41 38 or 39 or 40 or 41 or 42
42 (37) N6 (43)
44 Poststroke* [TIAB]
45 post*stroke [TIAB]
46
47 Isch#emi* attack*
48 Tia*1
49 Neurologic* deficit*
50 SAH
51 AVM
52 45 or…53
53 7 or 21 or 27 or 44 or 54
54 exp Cerebral Hemorrhage/
55 exp Cerebrovascular Accidents/
56 Stroke.mp
57 54 or 55 or 56
58 53 or 57

NHS EED

MeSH DESCRIPTOR stroke EXPLODE ALL TREES IN NHSEED

“Concept 2” = Health Economics

Search strategy derived from NHS CRD guidelines for economic study searching.

	MEDLINE	EMBASE
1	Economics/	Economics/
2	Exp economics	Exp economics
3	hospital/	hospital/
4	Exp economics, medical/	Exp economics, medical/
5	Economics, nursing/	Economics, nursing/
6	Economics, pharmaceutical/	Economics, pharmaceutical/
7	exp Healthcare costs/	exp health resources
8	Health care economics, organizations/	Health care economics, organizations/
9	Health care costs/	Health care costs/
10	Direct service costs/	Direct service costs/
11	Health expenditures/	Health expenditures/
12	Capital expenditures/	Capital expenditures/
13	Hospital costs/	Hospital costs/
14	Value of life/	Value of life/
15	economic$.ti,ab,kf	economic$.ti,ab,kw
16	pharmacoeconomic$.ti,ab,kf	pharmacoeconomic$.ti,ab,kw
17	price$.ti,ab,kf	price$.ti,ab,kw
18	pricing.ti,ab,kf	pricing.ti,ab,kw
19	Cost of Illness/	Cost of Illness/
20	Cost estimation/	Cost estimation/
21	"costs AND cost analysis"/	"costs AND cost analysis"/
22	Cost allocation/	Cost allocation/
23	Cost-benefit analysis/	Cost-benefit analysis/
24	Cost control/	Cost control/
25	Cost savings/	Cost savings/
26	Cost of illness/	Cost of illness/
27	Cost sharing/	Cost sharing/
28	Cost study/	Cost study/
29	Cost estimation/	Cost estimation/
30	Cost utility/	Cost utility/
31	Cost saving/	Cost saving/
32	Exp "fees and charges"/	Exp "fees and charges"/
33	Exp budgets/	Exp budgets/
34	(low adj3 cost).ti,ab,kf	(low adj3 cost).ti,ab,kw
35	(high adj3 cost).ti,ab,kf	(high adj3 cost).ti,ab,kw
36	(cost adj3 estimate$).ti,ab,kf	(cost adj3 estimate$).ti,ab,kw
37	(cost adj3 variable).ti,ab,kf	(cost adj3 variable).ti,ab,kw
38	(unit adj3 cost$).ti,ab,kf	(unit adj3 cost$).ti,ab,kw
39	(health?care adj4 cost$).ti,ab,kf	(health?care adj4 cost$).ti,ab,kw
40	fiscal.ti,ab,kf	fiscal.ti,ab,kw

41	funding.ti,ab,kf	funding.ti,ab,kw
42	financial.ti,ab,kf	financial.ti,ab,kw
43	finance.ti,ab,kf	finance.ti,ab,kw
44	(expenditure$ NOT energy) .ti,ab,kf	(expenditure$ NOT energy) .ti,ab,kw
45	"DALY".ti,ab,kf	"DALY" .ti,ab,kw
46	"disability adjusted life year$".ti,ab,kf	"disability adjusted life year$".ti,ab,kw
47	"quality adjusted life year$".ti,ab,kf	"quality adjusted life year$".ti,ab,kw
48	"QALY".ti,ab,kf	"QALY".ti,ab,kw
49	"economic appraisal" . ti,ab,kf	"economic appraisal" . ti,ab,kw
50	"economic analysis" . ti,ab,kf	"economic analysis" . ti,ab,kw
51	"economic evaluation$" . ti,ab,kf	"economic evaluation$" . ti,ab,kw
52	(resource$1 or utilisation or utilization).ti,ab,kf.	(resource$1 or utilisation or utilization).ti,ab,kw
53		
54	(bed adj3 day$1).ti,ab,kf.	(bed adj3 day$1).ti,ab,kw
56	Ec.fs.	pe.fs.
57	Office Visits/sn, td, ut	*ambulatory care/ and (exp *statistics/ or *health care utilization/)
58	(frequency or return or readmission$ or script$1 or prescription$ or prescribing).ti,ab.	(frequency or return or readmission$ or script$1 or prescription$ or prescribing).ti,ab.
59	1 OR 2…. For all searches	1 OR 2…. For all searches

CINAHL (EBSCO)

1	(MH "Economics+")
2	(MH "Financial Management+")
3	(MH "Financial Support+")
4	(MH "Financing, Organized+")
5	(MH"business+")
6	Or/2-5
7	1 not 6
8	MM Health resource allocation
9	MM Health resource utilization
10	8 or 9
11	7 or 10
12	(cost or costs or economic* or pharmacoeconomic* or price* or pricing*).[TIAB]
13	11 or 12
14	PT Editorial
15	PT Letter
16	PT News
17	Or/14-16
18	13 not 17
19	SO Cochrane library
20	AU Anonymous
21	20 not (21 or 22)

PsycINFO (EBSCO)

1	DE "Economics" OR DE "Behavioral Economics" OR DE "Evolutionary Economics" OR DE "Health Care Economics" OR DE "Neuroeconomics" OR DE "Pharmacoeconomics"
2	DE "Health Care Costs" OR DE "Health Care Economics" OR DE "Health Care Utilization"
3	DE "Costs and Cost Analysis"
4	DE “business”
5	1-3/OR
6	5 not 4
7	(cost or costs or economic* or pharmacoeconomic* or price* or pricing*).[TIAB]
8	6 or 7
9	PT Editorial
10	PT Letter
11	PT News
12	Or/9-11
13	8 not 12
14	SO Cochrane library
15	AU Anonymous
16	13 not (14 or 15)

Concept 3: Pre-coordination in information retrieval

MEDLINE: Stroke/ec [economics]

EMBASE: cerebrovascular accident/dm [disease management]

Linking Search strategies:

Concept 1: Stroke

Concept 2: Health economics

Concept 3: Pre-coordination in information retrieval

(Concept 1 ‘AND’ Concept 2) ‘OR’ Concept 3 = topic results

To exclude animal studies search was filtered further by:

Topic results ‘NOT’ (animals/ not humans/)

AND, OR, NOT = Boolean search operators.

Additional Search Limiters

OVID:

Limited to Abstracts/Structured Abstracts/Full text/ 2004-Current: Using OVID search filters

EBSCOhost:

PsycINFO: Linked Full Text; Published Date: 20040101-20150231; Peer Reviewed; Publication Type: All Journals; Population Group: Human; Exclude Dissertations

RESULTS

MEDLINE: 3081

EMBASE: 16470

Cinahl: 125

PsycINFO: 453
